# Supplementary material for: Drivers of Three Most Charismatic Mammalian Species Distribution across a Multiple-Use Tropical Forest Landscape of Sumatra, Indonesia
Source: Animals (Basel). 2022 Oct 10;12(19):2722. doi: 10.3390/ani12192722 (PMC9559281; doi:10.3390/ani12192722)
Supplement: Supplementary file 1 [file animals-12-02722-s001.zip › animals-1938412-supplementary.pdf]

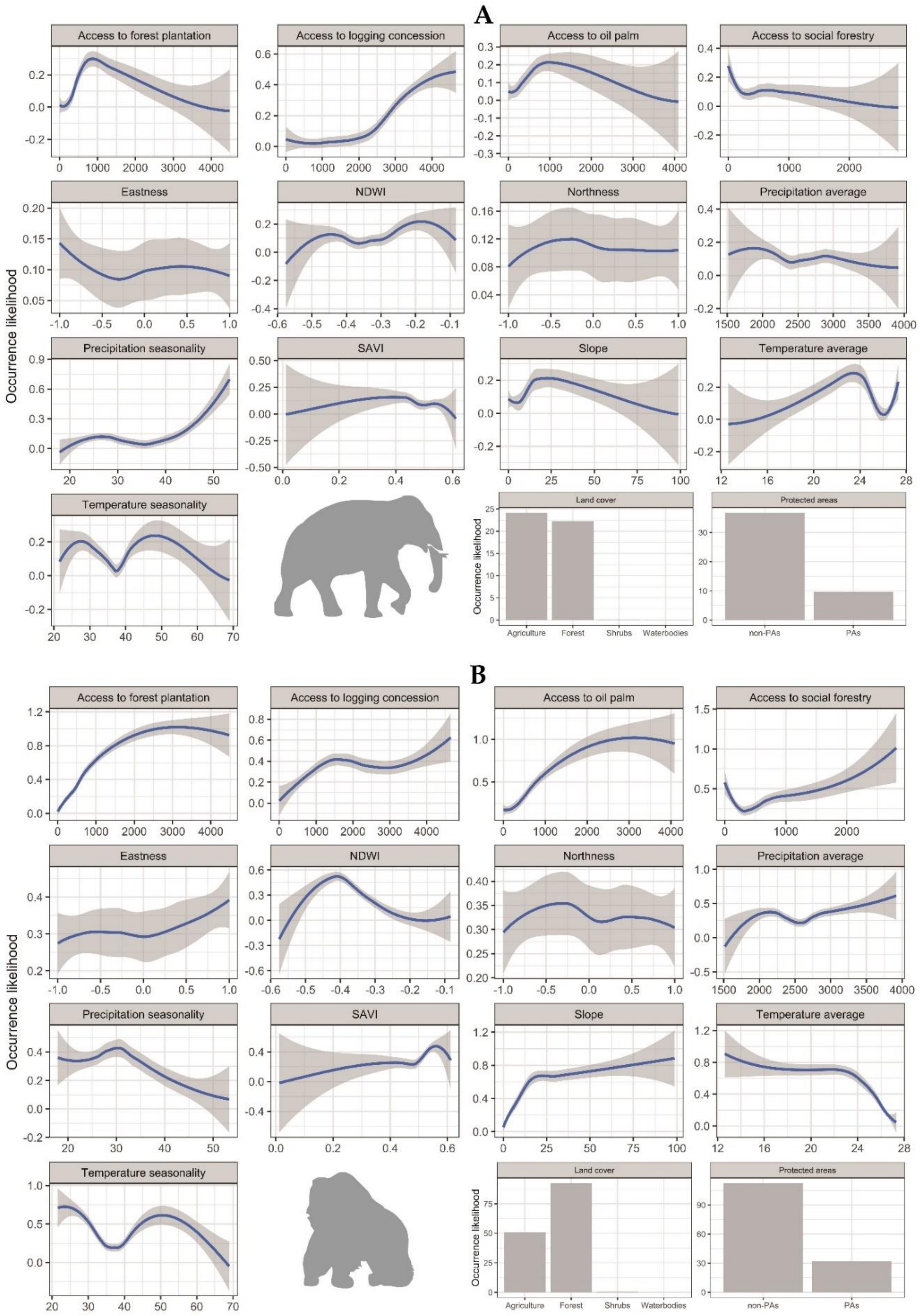

A
Access to forest plantation
Access to logging concession
Access to oil palm
Access to social forestry
Eastness
NDWI
Northness
Precipitation average
Precipitation seasonality
SAVI
Slope
Temperature average
Temperature seasonality
Land cover
Protected areas
Occurrence likelihood
Agriculture
Forest
Shrubs
Waterbodies
non-PAs
PAs
B
Access to forest plantation
Access to logging concession
Access to oil palm
Access to social forestry
Eastness
NDWI
Northness
Precipitation average
Precipitation seasonality
SAVI
Slope
Temperature average
Temperature seasonality
Land cover
Protected areas
Occurrence likelihood
Agriculture
Forest
Shrubs
Waterbodies
non-PAs
PAs

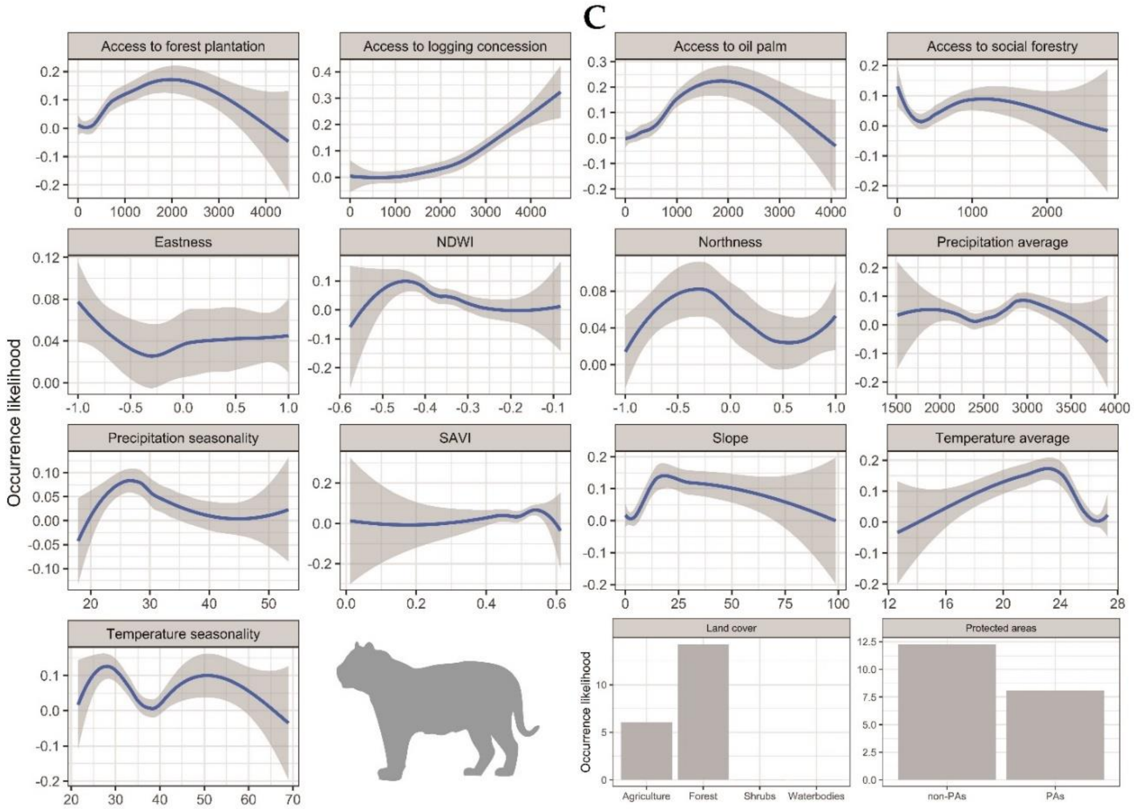

Figure S1. Response curves for each environmental layers of the best algorithm of *Elephas maximus sumatranus* (A), *Pongo abelii* (B), and *Panthera tigris sumatrae* (C).

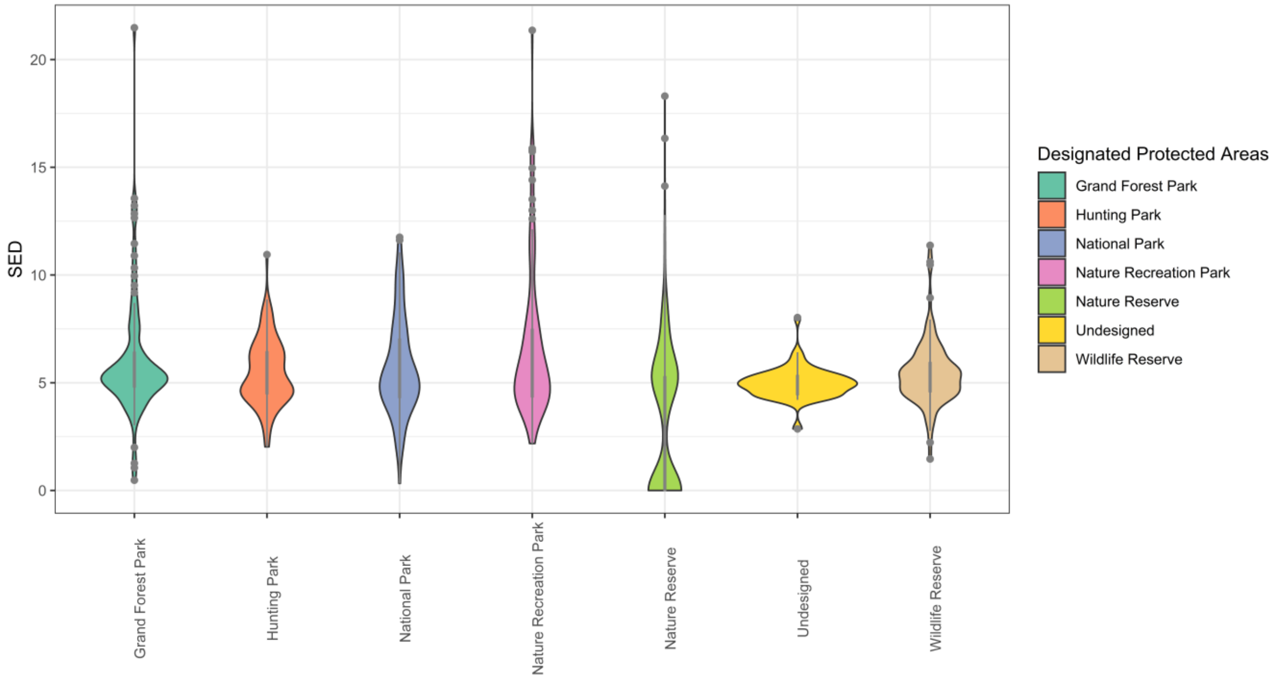

Figure S2. Climatic responses to the protected areas status.

Table S1. Description of 20 environmental variables that influence *Elephas maximus sumatranus*, *Pongo abelii*, and *Panthera tigris sumatrae* in Sumatran Island.

Group	Variables	Sources
Topographic	Slope	SRTM (Farr et al., 2007)
	Elevation	SRTM (Farr et al., 2007)
	Eastness	SRTM (Farr et al., 2007)
	Northness	SRTM (Farr et al., 2007)
	Aspect	SRTM (Farr et al., 2007)
Biophysics	Enhanced Vegetation Index (EVI)	Landsat 8 (Huete et al., 2002)
	Normalized Difference Vegetation Index (NDVI)	Landsat 8 (Huete et al., 2002)
	Normalized Difference Wetness Index (NDWI)	Landsat 8 (Gao, 1996)
	Index-based Built-up Index (IBI)	Landsat 8 (Xu, 2008)
	Soil-Adjusted Vegetation Index (SAVI)	Landsat 8 (Huete, 1988)
	Protected areas	MoEF
Anthropogenic	Land cover	CCI-ESA (Li et al., 2018)
	Access to oil palm plantation	WRI
	Access to logging concession	MoEF
	Access to industrial plantation concession	MoEF
	Access to social forestry concession	MoEF
Climatic	Mean annual temperature	WorldClim v2.0 (Fick and Hijmans, 2017)
	Temperature seasonality	WorldClim v2.0 (Fick and Hijmans, 2017)
	Mean annual precipitation	WorldClim v2.0 (Fick and Hijmans, 2017)
	Precipitation seasonality	WorldClim v2.0 (Fick and Hijmans, 2017)

Table S2. Predictive performance of the models on the k-fold (k = 5) for each species using five different metrics of accuracy, show in mean of value ± SE – i.e., Area Under the ROC Curve (AUC), Kappa coefficient, True Skill Statistic (TSS), Jaccard Index, and Sørensen Index.

Species	Algorithm	AUC	Kappa	TSS	Jaccard	Sørensen
Elephas maximus sumatranus	BRT	0.85 ± 0.016	0.55 ± 0.035	0.55 ± 0.035	0.65 ± 0.028	0.79 ± 0.021
	MXD	0.81 ± 0.015	0.48 ± 0.050	0.48 ± 0.050	0.60 ± 0.014	0.75 ± 0.011
	RDF	0.88 ± 0.009	0.60 ± 0.032	0.60 ± 0.032	0.70 ± 0.020	0.82 ± 0.014
	SVM	0.80 ± 0.021	0.46 ± 0.050	0.46 ± 0.050	0.60 ± 0.024	0.75 ± 0.018
	ENS	0.88 ± 0.010	0.59 ± 0.037	0.59 ± 0.037	0.68 ± 0.015	0.81 ± 0.011
Pongo abelii	BRT	0.96 ± 0.005	0.84 ± 0.019	0.84 ± 0.019	0.86 ± 0.016	0.92 ± 0.009
	MXD	0.95 ± 0.007	0.85 ± 0.015	0.85 ± 0.015	0.87 ± 0.014	0.93 ± 0.008
	RDF	0.97 ± 0.004	0.86 ± 0.011	0.86 ± 0.011	0.87 ± 0.008	0.93 ± 0.004
	SVM	0.95 ± 0.009	0.81 ± 0.020	0.81 ± 0.020	0.84 ± 0.016	0.91 ± 0.010
	ENS	0.96 ± 0.007	0.86 ± 0.016	0.86 ± 0.016	0.88 ± 0.014	0.93 ± 0.008
Panthera tigris sumatrae	BRT	0.89 ± 0.022	0.64 ± 0.031	0.64 ± 0.031	0.72 ± 0.018	0.84 ± 0.012
	MXD	0.88 ± 0.021	0.62 ± 0.060	0.62 ± 0.060	0.71 ± 0.031	0.83 ± 0.021
	RDF	0.89 ± 0.019	0.65 ± 0.032	0.65 ± 0.032	0.73 ± 0.023	0.84 ± 0.015
	SVM	0.88 ± 0.021	0.66 ± 0.046	0.56 ± 0.046	0.73 ± 0.027	0.84 ± 0.017
	ENS	0.90 ± 0.020	0.67 ± 0.051	0.67 ± 0.051	0.74 ± 0.032	0.85 ± 0.021

Table S3. List of predicted suitable landscapes and land use characteristics with species record in protected and non-protected areas landscape for *Elephas maximus sumatranus*, *Pongo abelii*, and *Panthera tigris sumatrae*.

Land cover types	Protected areas (ha)			Non-protected areas (ha)		
	Elephas maximus sumatranus	*Pongo abelii*	*Panthera tigris sumatrae*	*Elephas maximus sumatranus*	*Pongo abelii*	*Panthera tigris sumatrae*
Agriculture	263,932.41	15,023.54	254,502.44	251,1338.80	97,922.73	208,159.18
Forest	737,755.48	714,576.74	3,417,720.73	2,200,906.99	1,813,047.52	4,111,219.92
Shrubs	3059.93	57.38	3149.95	34,727.81	1237.68	7650.52
Urban areas	185.46	0	156.55	1309.66	0	111.76
Waterbodies	310.70	115.86	976.67	21,823.15	604.24	1892.85

Table S4. List of predicted suitable landscapes for three charismatics species record in protected area landscape of Sumatran Island.

Province	Protected area	Designation	Size of area (ha)
Elephas maximus sumatranus			
Nanggroe Aceh Darussalam	Gunung Leuser	National Park	62,003.06
	Hutan Pinus/ Janthoi	Nature Reserve	6570.98
	Janthoi	Nature Recreation Park	241.71
	Lingga Isaq	Hunting Park	23,969.01
	Pocut Meurah Intan	Grand Forest Park	1640.99
	Raflessia I/II Serbojadi	Nature Reserve	300.46
North Sumatra	Gunung Leuser	National Park	25,308.61
Riau	Balai Raja	Wildlife Reserve	2230.29
	Bukit Bungkuk	Nature Reserve	380.34
	Bukit Tiga Puluh	National Park	41,949.84
	Giam Siak Kecil	Wildlife Reserve	2757.54
	PLG Sebanga	Wildlife Reserve	1657.15
	Sultan Syarif Kasim (Minas)	Grand Forest Park	2005.89
	Tesso Nilo	National Park	57,620.55
Jambi	Bukit Tiga Puluh	National Park	7517.30
	Buluh Hitam	Undesigned	456.03
	Kerinci Seblat	National Park	32,890.91
	Sekitar Tanjung (Orang Kayo Hitam)	Grand Forest Park	158.78
Bengkulu	Bukit Barisan Selatan	National Park	52,202.80
	Kerinci Seblat	National Park	106,343.22
	Seblat	Nature Recreation Park	4306.84
	Way Hawang	Nature Recreation Park	15.00
South Sumatra	Bentayan	Wildlife Reserve	12,404.50
	Bukit Barisan Selatan	National Park	13.43
	Dangku	Wildlife Reserve	13,195.77
	Gunung Raya	Wildlife Reserve	27,996.59

	Kerinci Seblat	National Park	67,339.77
	Padang Sugihan	Wildlife Reserve	62,145.31
Lampung	Bukit Barisan Selatan	National Park	238,996.04
	Gunung Raya	Wildlife Reserve	2265.55
	Rawa Kandis	Undesigned	1058.19
	Wan Abdul Rahman	Grand Forest Park	20,493.12
	Way Kambas	National Park	126,808.41
Pongo abelii			
Nanggroe Aceh Darussalam	Gunung Leuser	National Park	429,527.61
	Hutan Pinus/ Janthoi	Nature Reserve	13,620.44
	Janthoi	Nature Recreation Park	1004.44
	Kuta Malaka	Nature Recreation Park	66.04
	Lingga Isaq	Hunting Park	57,493.44
	Pocut Meurah Intan	Grand Forest Park	4299.94
	Raflessia I/II Serbojadi	Nature Reserve	308.86
	Rawa Singkil	Wildlife Reserve	78,421.02
North Sumatra	Gunung Leuser	National Park	141,694.32
	Siranggas	Wildlife Reserve	3337.40
Panthera tigris sumatrae			
Nanggroe Aceh Darussalam	Gunung Leuser	National Park	564,580.59
	Hutan Pinus/ Janthoi	Nature Reserve	15,651.48
	Lingga Isaq	Hunting Park	36,416.16
	Rawa Singkil	Wildlife Reserve	1505.50
	Subulussalam	Grand Forest Park	1016.36
	Pocut Meurah Intan	Grand Forest Park	4203.83
	Serbojadi	Nature Reserve	34.98
North Sumatra	Barumun	Wildlife Reserve	37,428.98
	Batang Gadis	National Park	59,717.55
	Bukit Barisan	Grand Forest Park	26,792.69
	Dolok Saut	Nature Reserve	126.74
	Dolok Sibual-Buali	Nature Reserve	4023.54
	Dolok Sipirok	Nature Reserve	7006.49
	Dolok Surungan	Wildlife Reserve	15,193.31
	Dolok Tinggi Raja	Nature Reserve	129.97
	Gunung Leuser	Grand Forest Park	176,764.46
	Karang Gading Timur Laut	Nature Reserve	654.76
	Lau Debuk Debuk	Nature Reserve	2.89
	Lubuk Raya	Nature Reserve	1806.39
	Sibolangit	Nature Reserve	25.86
	Sicikeh-Cikeh	Nature Reserve	470.39
	Siranggas	Wildlife Reserve	5422.98

West Sumatra	Air Putih	Nature Recreation Park	7526.46
	Arau Hilir dan Air Terusan	Wildlife Reserve	87,040.00
	Batang Gadis	National Park	96.71
	Batang Pangean I	Nature Reserve	12,358.96
	Batang Pangean II	Nature Reserve	29,229.92
	Bukit Rimbang Bukit Baling	Wildlife Reserve	1676.24
	Gunung Marapi	Nature Recreation Park	5842.76
	Gunung Sago Malintang	Nature Recreation Park	3797.51
	Kerinci Seblat	National Park	287,119.31
	Lembah Anai	Nature Reserve	305.74
	Lembah Harau	Nature Recreation Park	125.17
	Malampah Alahan Panjang	Wildlife Reserve	36,840.11
	Maninjau	Nature Reserve	17,465.82
	Mega Mendung	Nature Recreation Park	4.15
	Rimbo Panti	Nature Recreation Park	2876.25
	Singgalang Tandikat	Nature Recreation Park	6806.69
Riau	Air Putih	Nature Recreation Park	682.92
	Balai Raja	Wildlife Reserve	397.41
	Bukit Batu	Wildlife Reserve	20,327.38
	Bukit Bungkuk	Nature Reserve	11,469.09
	Bukit Rimbang Bukit Baling	Wildlife Reserve	132,001.38
	Bukit Tiga Puluh	National Park	101,807.68
	Buluh Cina	Nature Recreation Park	28,354.49
	Giam Siak Kecil	Nature Reserve	52,693.55
	Kerumutan	Wildlife Reserve	91,604.88
	Mahato	Nature Reserve	821.69
	Pulau Berkeh	Nature Reserve	4116.68
	Sultan Syarif Kasim/ Minas	Grand Forest Park	496.69
	Tasik Belat	Wildlife Reserve	1670.69
	Tasik Besar-Tasik Metas	Wildlife Reserve	3911.54
	Tasik Serkap-Tasik Sarang Burung	Wildlife Reserve	7072.61
	Tesso Nilo	National Park	59,255.58
Jambi	Berbak	National Park	130,226.84
	Bukit Dua Belas	National Park	40,992.56
	Bukit Tiga Puluh	National Park	37,821.77
	Buluh Hitam	Nature Reserve	256.98
	Hutan Bakau Pantai Timur	Nature Reserve	571.28
	Kerinci Seblat	National Park	375,138.91
	Sembilang	National Park	11,035.34
Bengkulu	Air Ketebat Danau Tes	Nature Recreation Park	1653.76
	Air Seblat	Nature Reserve	7334.53

	Bukit Barisan Selatan	National Park	63,367.40
	Bukit Kaba	Nature Recreation Park	12,223.67
	Bukit Rabang - Gluguran	Grand Forest Park	417.33
	Danau TES	Nature Recreation Park	345.49
	Kerinci Seblat	National Park	308,007.31
	Rajo Lelo (Pungguk Menakat)	Nature Recreation Park	754.44
	Semidang Bukit Kabu	Hunting Park	8634.06
	Way Hawang	Nature Recreation Park	4.10
South Sumatra	Bukit Barisan Selatan	National Park	44.70
	Dangku	Wildlife Reserve	12,178.68
	Gumai Tebing Tinggi	Wildlife Reserve	45,859.16
	Gunung Raya	Wildlife Reserve	22,009.71
	Isau-isau Pasemah	Wildlife Reserve	15,904.70
	Kerinci Seblat	National Park	218,501.18
	Sembilang	National Park	33,133.72
Lampung	Bukit Barisan Selatan	National Park	249,685.30
	Gunung Raya	Wildlife Reserve	926.74
	Wan Abdul Rahman	Grand Forest Park	14,694.98
	Way Kambas	National Park	90,009.79

Table S5. Geographical range of suitable landscape for *Elephas maximus sumatranus*, *Pongo abelii*, and *Panthera tigris sumatrae* in Sumatran Island in protected areas (PA), non-protected areas (non-PA), Forest plantation concession (FPC), Logging concessions (LC), Industrial oil palm plantation (IOPP), and Social Forestry Concession (SFC) (areas in ha).

Province	Total Area	PA (Total PA)	Non-PA (Total PA)	FPC (Total FPC)	LC (Total LC)	IOPP (Total IOPP)	SFC (Total SFC)
Elephas maximus sumatranus							
Nanggroe Aceh Darussalam	5,688,779.18	94,726.20 (856,277.48)	1,177,963.75 (4,832,501.71)	109,896.78 (213,575.66)	109,675.27 (116,781.43)	13,869.31 (292,826.25)	40,390.23 (110,772.61)
North Sumatra	7,198,584.10	25,308.61 (420,777.86)	125,715.22 (6,777,806.25)	79,111.77 (344,394.95)	0 (244,101.76)	923.14 (268,550.54)	12,248.62 (97,386.25)
Riau	8,955,130.82	108,601.62 (626,126.24)	782,322.75 (8,329,004.58)	453,808.94 (1,555,333.45)	0 (134,502.14)	60,184.97 (2,209,216.02)	10,260.86 (55,311.98)
Jambi	4,932,120.04	41,023.02 (731,159.01)	411,842.56 (4,200,961.03)	262,397.30 (675,871.04)	932.41 (56,157.77)	2958.61 (345,293.64)	32,236.60 (170,775.72)
Bengkulu	1,961,059.86	162,867.86 (444,476.78)	121,457.69 (1,516,583.08)	0 (0)	60,641.22 (64,766.07)	38,619.88 (120,474.92)	576,70 (22,332.69)
South Sumatra	8,719,635.93	183,095.38 (720,836.33)	1,106,098.45 (7,998,799.60)	574,842.08 (1,300,093.86)	0 (0)	48,534.23 (364,617.07)	1750.23 (76,430.07)
Lampung	3,369,982.92	389,621.31 (417,179.79)	1,044,705.98 (2,952,803.13)	14,227.43 (124,050.04)	0 (0)	29,822.40 (99,059.39)	82,359.10 (94,224.04)
Total	40,825,292.85	1,005,244.00 (4,216,833.49)	4,770,106.40 (36,608,459.38)	1,494,284.30 (4,213,319.00)	171,248.90 (616,309.17)	194,912.54 (3,700,037.83)	179,245.64 (806,479.00)
Pongo abelii							
Nanggroe Aceh Darussalam	5,688,779.18	584,741.79 (856,277.48)	1,820,374.76 (4,832,501.71)	90,665.93 (213,575.66)	67.38 (116,781.43)	21,531.87 (292,826.25)	61,671.04 (110,772.61)
North Sumatra	7,198,584.10	145,031.73 (420,777.86)	92,437.41 (6,777,806.25)	0 (344,394.95)	0 (244,101.76)	0 (268,550.54)	3966.61 (97,386.25)
Total	12,887,363.28	729,773.52 (1,277,055.34)	1,912,812.17 (11,610,307.96)	90,665.93 (557,970.61)	67.38 (360,883.19)	21,531.87 (561,376.79)	65,637.65 (208,158.86)
Panthera tigris sumatrae							
Nanggroe Aceh Darussalam	5,688,779.18	623,408.91 (856,277.48)	1,217,771.97 (4,832,501.71)	6507.05 (213,575.66)	75,654.95 (116,781.43)	40.31 (292,826.25)	28,317.24 (110,772.61)
North Sumatra	7,198,584.10	335,566.99 (420,777.86)	732,870.50 (6,777,806.25)	27,473.22 (344,394.95)	62,578.61 (244,101.76)	3676.21 (268,550.54)	9113.32 (97,386.25)
West Sumatra	4,180,539.16	499,111.79 (763,121.77)	643,113.66 (3,417,417.38)	218.59 (63,591.09)	36,922.51 (181,002.25)	105.52 (208,154.05)	61,033.71 (205,239.82)

Riau	8,955,130.82	516,684.26 (626,126.24)	703,037.08 (8,329,004.58)	294,501.39 (1,555,333.45)	91,239.45 (134,502.14)	5034.30 (2,209,216.02)	8848.65 (55,311.98)
Jambi	4,932,120.04	596,043.68 (731,159.01)	198,064.63 (4,200,961.03)	7832.60 (675,871.04)	21,240.61 (56,157.77)	0 (345,293.64)	42,487.49 (170,775.72)
Bengkulu	1,961,059.86	402,742.07 (444,476.78)	394,302.57 (1,516,583.08)	0 (0)	27,935.06 (64,766.07)	28,820.25 (120,474.92)	2738.29 (22,332.69)
South Sumatra	8,719,635.93	347,631.86 (720,836.33)	223,969.97 (7,998,799.60)	8456.73 (1,300,093.86)	0 (0)	0 (364,617.07)	12,520.94 (76,430.07)
Lampung	3,369,982.92	355,316.80 (417,179.79)	215,903.84 (2,952,803.13)	0 (124,050.04)	0 (0)	911.54 (99,059.39)	14,066.00 (94,224.04)
Total	45,005,832.01	3,676,506.36 (4,979,955.26)	4,329,034.23 (40,025,876.76)	344,989.58 (4,276,910.09)	315,571.19 (797,311.42)	38,588.13 (3,908,191.88)	179,125.64 (832,473.18)
